# Effects of Prehabilitation on Functional Capacity in Aged Patients Undergoing Cardiothoracic Surgeries: A Systematic Review

**DOI:** 10.3390/healthcare9111602

**Published:** 2021-11-22

**Authors:** Damián Fernández-Costa, Juan Gómez-Salgado, Andrés Castillejo del Río, Álvaro Borrallo-Riego, María Dolores Guerra-Martín

**Affiliations:** 1 Primary Care Urgency Service, Huelva-Costa Device, 21003 Huelva, Spain; damianfernandezz98@gmail.com; 2 Sociology, Social Work and Public Health Department, University of Huelva, 21007 Huelva, Spain; salgado@uhu.es; 3 Postgraduate Programme on Safety and Health, Universidad Espíritu Santo, Samborondón 092301, Ecuador; 4 Nursing School, University of Huelva, 21007 Huelva, Spain; ancasdel16@gmail.com; 5 Nursing Department, University of Seville, 41009 Seville, Spain; aborrallo@us.es

**Keywords:** aged, cardiorespiratory fitness, functional status, preoperative exercise, thoracic surgery

## Abstract

Background: an increasing number of advanced age patients are considered for cardiothoracic surgeries. Prehabilitation optimizes the patients’ functional capacity and physiological reserve. However, the effectiveness of prehabilitation on physical functioning and postoperative recovery in the scope of cardiothoracic surgery is still uncertain. Objective: to assess the effectiveness of prehabilitation on pre- and/or postoperative functional capacity and physiological reserve in aged patients that are considered for cardiothoracic surgeries. Methods: this systematic review was registered in PROSPERO (CRD42021247117). The searches were conducted in PubMed, Web of Science, Scopus, and Cochrane CENTRAL until 18 April 2021. Randomized clinical trials that compared different prehabilitation strategies with usual care on the pre- and-postoperative results in aged patients undergoing cardiothoracic surgeries were included. Methodological quality was assessed by means of the Jadad scale, and the effectiveness of the interventions according to the Consensus on Therapeutic Exercise Training. Results: nine studies with 876 participants aged from 64 to 71.5 years old were included. Risk of bias was moderate due to the absence of double-blinding. The content of the interventions (multimodal prehabilitation n = 3; based on physical exercises n = 6) and the result measures presented wide variation, which hindered comparison across the studies. In general, the trials with better therapeutic quality (n = 6) reported more significant improvements in physical functioning, cardiorespiratory capacity, and in the postoperative results in the participants under-going prehabilitation. Conclusions: prehabilitation seems to improve functional capacity and postoperative recovery in aged patients undergoing cardiothoracic surgeries. However, due to the significant heterogeneity and questionable quality of the trials, both the effectiveness of prehabilitation and the optimum content are still to be determined.

## 1. Introduction

Due to demographic aging, an ever-increasing number of advanced age patients (due to strategic reasons, different professional associations establish 60-years-old as the cutoff point) and of high-risk patients are considered for the performance of important cardiothoracic surgeries [1,2]. During the waiting period, the loss of functional capacity produced in these patients due to the combination of aging, frailty risk, and the underlying base pathologies exert a deep impact on physical functioning and health-related quality of life after the surgery [3,4,5].

Functional capacity is herein defined as physical and mental fitness to perform the basic activities of daily living and which allows the patients to face stressors such as the disease or the surgery [6,7]. In particular, slow gait according to the 6-min walk test, low cardiorespiratory fitness and deficient pulmonary function are the main preoperative functional parameters associated with prolonged hospitalizations, development of postoperative complications, and increased morbidity and mortality [8,9,10]. Therefore, the interventions aimed at improving these conditions, such as preoperative rehabilitation (prehabilitation), gradually gained greater recognition [5,11].

The objective of prehabilitation is to optimize the patients’ functional capacity and physiological reserve between indication and performance of the thoracic surgery, to properly prepare them for the surgical aggression. It generally includes a physical training program or multimodal therapy [5,12]. When compared to that of usual preoperative care and conventional rehabilitation [13], it was observed that prehabilitation programs seem to reduce hospitalization time and accelerate return to the functional state prior to surgery in various surgical contexts [14,15].

However, in the scope of cardiothoracic surgery, the effectiveness of prehabilitation on physical functioning and postoperative recovery is still uncertain, especially in the case of aged patients [5]. Up to date, several systematic reviews were published highlighting the heterogeneity of interventions and results [16,17,18,19], but none of them focused exclusively on high-risk patients or assessed the programs’ content according to standardized guidelines [20], despite the latest international recommendations about a new scale to assess the therapeutic quality of the prehabilitation protocols [21].

Sadeghi et al. [22] realized a review to examine the existing literature to address the virtual tools currently used in the areas of preoperative surgical planning, intraoperative guidance, and postoperative management in the field of cardiothoracic surgery. Although these online applications were not widely used in cardiothoracic surgery, they already showed great potential in providing diagnostics, preoperative planning, intraoperative guidance, and postoperative treatment. However, further research is required to develop the application of these techniques in cardiothoracic surgery. This lack of evidence on the subject justifies the pertinence of this systematic review, with the aim to analyze effects of prehabilitation on functional capacity in aged patients undergoing cardiothoracic surgeries. The PICOS questionnaire was: (P) Aged patients undergoing elective cardiothoracic surgery; (I) prehabilitation program (physical exercise-based therapy or multimodal prehabilitation); (C): compared with usual care; (O): improves in functional status (physical condition and health-related quality of life), physiological reserve (Cardiorespiratory Fitness), and clinical outcomes (postoperative complications, mortality and length of stay); (S): randomized clinical trials.

## 2. Materials and Methods

### 2.1. Study Design

A peer systematic review was carried out, following the Preferred Reporting Items for Systematic reviews and Meta-Analyses (PRISMA) statement and the Cochrane manual for intervention systematic reviews, which ensure the conduction of complete and rigorous reviews [23,24]. The study protocol was registered in PROSPERO (CRD42021247117) before selection and data extraction.

### 2.2. Search Strategies and Selection Criteria

The following databases were consulted (without date restrictions) to identify relevant studies: MEDLINE (via PubMed), Web of Science, Scopus, and Cochrane Central Register of Controlled Trials until 18 April 2021. As a secondary strategy, the reference lists of the relevant reviews and of the clinical trials included were consulted and ClinicalTrials.gov was explored to assess unpublished or ongoing trials. Two reviewers conducted the searches independently from the keywords identified on the theme. The detailed search strategy for each database can be consulted in Table S1.

The study selection criteria were as follows: (1) high-risk aged patients (80 and over) with functional limitations in the absence of physical disability or comorbidities that contraindicate physical exercise (unstable heart diseases, dementia, etc.); (2) waiting for any scheduled elective cardiothoracic surgery; (3) randomized clinical trials in English, Spanish, or Portuguese, (4) assessing the therapeutic effectiveness of a prehabilitation program in relation to a control group on functional capacity and/or physiological reserve before and/or after the surgery; and (5) according to validated criteria (for example: Peak VO2, FEV1, etc.). However, the studies were not eligible if: (1) they included relatively healthy patients (according to an objective assessment of the preoperative health status), (2) the therapy lasted less than one week, (3) relevant information about the prehabilitation content was missing and integrity of the intervention could not be ensured, or (4) the results of interest were not measured or reported.

### 2.3. Data Analysis and Assessment of Article Quality

Two reviewers selected the studies independently according to their titles and abstracts and, subsequently, as per the pre-established eligibility criteria. Any and all disagreements about study selection were resolved through consensus with a third author.

Subsequently, two reviewers separately extracted the data from the selected articles, and three authors participated in the discussion and synthesis of the results. A registration form was designed following the indications set forth in the Cochrane Manual [24], detailing author(s) and date, sample, description of the interventions’ content according to the principles set forth by the American College of Sports Medicine/American Heart Association [25]; (4) result measures, and (5) main findings.

The methodological quality of the included studies was assessed by two independent reviewers using the Jadad scale [26]. The methodological quality was categorized as low (<3), acceptable (3), good (4), and excellent (5), based on a maximum score of 5. Disagreements about the risk of bias were resolved by consulting the third reviewer.

To determine the integrity of the nonpharmacological interventions, one reviewer separately evaluated the therapeutic validity of the prehabilitation programs using the Consensus on Therapeutic Exercise Training scale. The scale includes nine yes-no questions, encompassing five categories, namely: selection of patients, selection of the therapist and environment, justification, content, and adherence. Therapeutic validity was categorized as low (0–5/9) or as high (≥6/9), according to the cutoff points proposed [21].

## 3. Results

### 3.1. Presentation of the Studies

**Figure 1:**
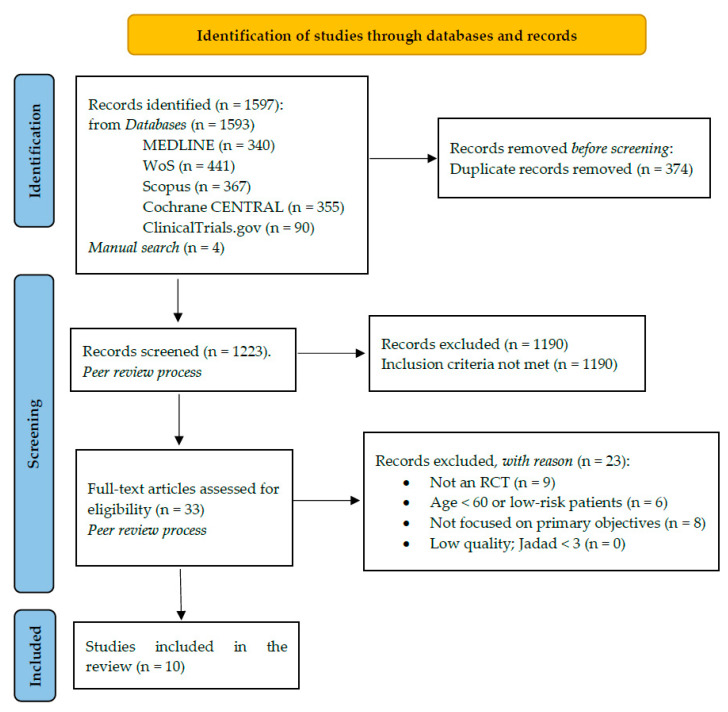
Study selection PRISMA 2020 Flowchart.

A total of nine studies (with 876 participants aged from 64 to 71.5 years old) met the inclusion criteria and were included (Figure 1).

Five studies focused on patients awaiting pulmonary resection due to nonsmall cell lung cancer [27,28,29,30,31]; two studies with patients subjected to esophagectomy [32,33], and the remaining two with patients considered for coronary artery bypass grafting [34]. All the studies included high-risk patients according to different objective classifications: six studies due to physical unfitness according to a cardiopulmonary resistance [27,35] or respiratory function [28,29,30,31] test, and five based on unfavorable scores in the scales used to assess the preoperative health status [28,31,32,33,34]. The characteristics of the studies are presented in Table S2.

### 3.2. Quality Assessment

The nine trials presented an “acceptable” risk of bias (see Table S3). All the included trials reported suitably regarding random sequence generation and concealment. No modality for control treatment was used in the studies due to the nature of the intervention, thus precluding double-blinding. In most of the trials, the evaluators and therapists remained masked, and two studies also blinded the statisticians and lead researchers, although they did not describe the process [28,33]. All the trials reported suitably on the follow-up losses and took into consideration the number of abandonments for data analysis. The sample size of the studies ranged from 22 to 238 participants. In four trials, statistical power was limited by the reduced sample size [29,31,33,35].

### 3.3. Thematic Analysis

Four domains of experience were inductively developed from the analysis.

#### 3.3.1. Therapeutic Validity

Six of the nine studies presented high therapeutic validity [27,28,31,33,34,35] (see Table S4). All the trials made a correct selection of the participants, by including high-risk patients after an initial and exhaustive assessment of their health status. Except for Morano et al. [29], eight studies considered the program’s follow-up by trained staff. In six trials, all the sessions were supervised and monitored by the therapists in the hospital [27,28,30,31] and community [34,35] rehabilitation rooms. The other two studies proposed a nonsupervised home program. These patients underwent a first in-person training session and, subsequently, follow-up was conducted with weekly phone calls and a registration book to assess issues related to compliance with the program [32,33].

The prehabilitation programs presented wide variation. All the studies included a physical training program, but only three assessed a multimodal program: one RCT included nutritional therapy [33] and another two, educational and motivational sessions [30,35]. Regarding prehabilitation with physical exercises, heterogeneity was observed in the programs’ duration (1 study: 1 week, 6 studies: 2–4 weeks, 2 studies: >5 weeks), frequency (2–5 times a week, daily) and physical training modalities. Guinan et al. [32] proposed an inspiratory muscle training program, whereas the remaining eight studies included aerobic training as a common denominator: two studies focused on unimodal aerobic training [27,35], two trials on aerobic training with inspiratory muscle training [28,30], one with muscle strengthening [33] and three, according to multicomponent training [29,31,34]. The main proposal was moderate-intensity aerobic training, or with High-Intensity Intervals (see Table S2). In general, seven studies adapted the programs’ content to the patients’ physical condition according to specific terms (Borg dyspnea scale, Peak VO2, heart rate, etc.). However, Morano et al. [29] and Vagvolgyi et al. [30] did not report on the sessions’ length and frequency, respectively.

A positive association is established between content customization and better compliance rate: of the five studies that adapted the sessions to the participants’ personal factors, and which adjusted intensity according to individual progress along the study [27,28,31,33,35], 80% obtained good adherence rates (with an average of 72.35%) [27,28,31,33].

#### 3.3.2. Functional Capacity

Despite the heterogeneity of the result measures, all the studies assessed 6-min walk test, and 5 out of 9 found effects on the covered distances after prehabilitation. In the coronary artery bypass grafting context, Steinmetz et al. [34] showed that an incremental-length high-intensity interval training program combined with inspiratory muscle training three times a week was associated with a significant improvement in functional capacity three weeks after the surgery (6-min walk test: +50.5 m; Time-up and Go test: −0.5 s; *p* < 0.001). Minnella et al. [33] found similar results in the covered distance for patients subjected to esophagectomy (pre-6-min walk test: +36.9 m; post-6-min walk test: +15.4 m; *p* < 0.001). Other functional capacity measures included strength tests for the upper and lower limbs [29,31] and frailty assessment according to different scales [35]. In this study, the prehabilitation regime only showed significant effects on the frailty, and 6-min walk test in those patients that complied with all the scheduled sessions (28.6%, n = 4) [35].

Five studies assessed health-related quality of life based on different questionnaires. Two studies made use of SF-36 with contradictory results [29,31], one study used EORTC-QLQ-C30 and the EORTC LC-13 scale for dyspnea [28], one study resorted to the questionnaire developed by the Medical Research Council [30] and another to the McNew scale for the cardiac population [34]. Although three out of five trials presented good therapeutic validity, only in the RCT by Sebio et al. [31] did a therapy with multicomponent exercises exert partial effects on physical functioning before and after the surgery (after SF-36, physical component: +4.3 vs. −4.8 points, *p* = 0.001).

#### 3.3.3. Physiological Reserve

Two studies evaluated Peak VO2 and peak aerobic work power (Peakw) [27,34], whereas four studies assessed physical activity level according to different objective measures: Guinan et al. [32] according to the number of steps walked/day and to the minutes of activity/day, and another three studies according to the sustained time during a cardiopulmonary effort test (Vagvolgyi et al. also measured the distance covered in kilometers and power in Watts) [30,31,35]. All the trials in which prehabilitation presented significant differences had good therapeutic validity.

Three studies assessed pulmonary function and that of the inspiratory muscles, although only one trial obtained beneficial effects: Lai et al. [28] showed a difference between the means of 21 L/min in Peak Expiratory Flow (95% CI: 7.2–34.8 L/min; *p* = 0.003) in the patients who underwent one-week prehabilitation therapy at full hospitalization regime. In the case of patients with chronic obstructive pulmonary diseases subjected to pulmonary resection, FEV1 and forced vital capacity improved before the surgery and after pulmonary rehabilitation, although there were no differences in relation to the control group, which underwent postoperative pulmonary rehabilitation [30].

#### 3.3.4. Secondary Results

Three trials analyzed the incidence of postoperative complications: Minnella et al. [33] used the Cavien–Dindo classification system and the Comprehensive Complication Index, Sebio et al. [31] resorted to the Melbourne group scale, and Lai et al. [28] used their own parameters to define the pulmonary complications and showed the most beneficial effects, by relating participation in prehabilitation with a reduction in the number of complications (9.8% vs. 28% in the control group; *p* = 0.019) and, with it, in hospitalization time (15.6 vs. 17.7 days, *p* = 0.023) and medical expenses (*p* = 0.023). Due to the reduced sample size in the rest of the trials, the incidence of complications was comparable, as well as the mortality and rehospitalization rates in the trial by Minnella et al. [33].

On the other hand, Valgovgyi et al. [30] made a correlation matrix for the changes in the 10 variables analyzed after a program with educational sessions, and found that four of them (gender, post-intervention FEV1, initial 6-min walk test value, and kilometers covered in the cycloergometry) could predict in 67% the number and severity of the postoperative complications of all the cases.

## 4. Discussion

The main finding of our review reinforces the value of the therapeutic validity, by showing a positive association between the studies with better therapeutic quality and the most significant improvements in functional capacity, level of physical exercise, pulmonary capacity, and perioperative clinical outcomes. However, our results must be interpreted with caution due to the variability observed regarding pre-enrollment of physical exercises and analyzed result measures. In addition, many trials focused exclusively on exercise-based prehabilitation, excluding other components of major clinical relevance such as nutritional and psychological support.

Prehabilitation has the potential to improve surgical outcomes in patients undergoing cardiothoracic surgery [17,19,36,37,38]. However, recently Kamarejah et al. [36] reported that the evidence from randomized studies remains weak owing to variation in prehabilitation regimes, limiting the assessment of current postoperative outcomes. Therefore, to discuss the possible clinical implications of our findings, we followed the latest recommendations published to evaluate the effectiveness of the therapeutic exercise programs. The key points include adequate selection of the participants, integration of customized programs, and their evaluation by means of result measures defined through consensus [20,21].

The 6-min walk test was recognized in the literature as a useful measure to identify patients with the greatest need for preoperative optimization [39]. In a prospective cohort study conducted with 882 patients subjected to coronary artery bypass grafting, aged individuals represented the subgroup that most improved the 6-min walk test results after a structured aerobic training [40]. These findings reinforce the evidence about a possible therapeutic window in these patients [4,11,41]. Our results also support this hypothesis, by carefully selecting advanced age patients with a deficient baseline physical condition, who greatly benefited from different types of prehabilitation, and because more than half of the trials included found specific benefits on the 6-min walk test. Jastrzebski et al. [42] realized a study on the physical fitness and mobility of patients with lung cancer after thoracic surgery to evaluate the influence of exercises on a stabilometric platform. The results indicated that the distance covered in the 6MWT significantly improved in the experimental group as well as the control group in all patients who completed the 2-week rehabilitation program.

Despite the suitable selection of high-risk patients, there is a need to assess the effectiveness of the different preoperative interventions through optimum result measures [20]. For example, although Peak VO2 is very useful for preenrollment in AT and progress evaluation in healthy and comorbid patients [37,43], in our context, several previous reviews have suggested that peak effort tests might not be the best measure to detect physiological changes related to the intervention [19,44]. However, an acknowledged limitation of the studies is that the intensity of the training sessions was not adapted to the participants’ baseline physical condition, which probably reduced the response capacity of this measure [44]. In this sense, this review can help to reach consensus about the validity of Peak VO2 and PeakW, as well as about the effectiveness of aerobic training in aged patients: Bathia & Kaiser and Steinmetz et al. proposed a prehabilitation program mainly based on two-week AT and measured the changes in aerobic capacity according to Peak VO2 and PeakW, although adapted according to subjective exhaustion in the training sessions and not to standardized criteria. This resulted in greater tolerance to the exercises, better compliance rates and, finally, in positive effects on cardiorespiratory fitness [27,34].

Another measure of interest can be health-related quality of life, as it turns out to be useful to report on pre-enrollment of the exercises and the required supervision level [20,45]. However, our findings are in line with previous reviews that highlight the heterogeneity of questionnaires reported by the studies, as well as the absence of important effects of the prehabilitation therapies on the health-related quality of life results after cardiothoracic surgery [17,19,38].

There seems to be more consensus about the benefits of prehabilitation on pre-and postoperative pulmonary function. A recent meta-analysis showed that different types of pre-operative exercise significantly increased FEV1 and FVC. In addition, pulmonary optimization reduced by 55% the incidence of pulmonary complications and by 4.83 days the total hospitalization time in aged patients with nonsmall cell lung cancer [19]. In our review, Lai et al. [28] presented similar results about pulmonary function and about the postoperative clinical outcomes after intensive aerobic training and incentive breathing. On the other hand, Guinan et al. [32] showed no benefits in the postoperative period after a home-based, 3-week inspiratory muscle training program, which is in opposition to other intensive inspiratory muscle training programs previously conducted and systematically developed [46].

Hospitalization time and prevalence of complications are usually reported for the outcomes after the surgery, although it is likely that they do not reflect the true effectiveness of prehabilitation, since the clinical impact varies according to the individual health status of each patient after undergoing some therapy [17,41]. Vagvolgyi et al. [30] identified a series of functional parameters that accurately predicted the severity of the complications, reasoning why it might be advantageous to combine a complication with its impact on this type of measure and, consequently, better reflect the therapeutic effects of prehabilitation. This was proven by previous meta-analyses, where pre-operative optimization of these parameters exerted therapeutic effects on a reduction in hospitalization time, incidence of complications, or admittance to intensive care units. However, certainty of the evidence was low due to the reduced sample sizes, to the variability of the results according to the type of surgery and to the scale used to assess the complications [17,37,38].

### 4.1. Strengths and Limitations

Due to the differences in the efficacy of prehabilitation across the trials, it was not suitable to conduct a meta-analysis or an analysis by subgroups; therefore, the optimum content, intensity, and length of the programs in aged patients waiting cardiothoracic surgeries are still to be determined. The main barriers were the insufficient sample sizes in some studies, the scarce number of trials that investigated a multimodal program, and the absence of uniform and customized prehabilitation protocols, evaluated by relevant result measures despite the latest evidence-based recommendations to ensure the integrity of the preoperative interventions [20,21].

Regarding risk of bias, none of the studies applied alternative blinding methods for the participants. Due to lack of double-blinding, these studies were more susceptible to conduction bias [47].

In an effort to improve our results, we must highlight some strengths. As nonpharmacological interventions generally need more data to describe the intervention, following the recommendations set forth in the Cochrane Manual, it was decided to provide reliable information about the environment, procedures, processes, and strategies to ensure compliance with prehabilitation (including the interventions in the control group) by means of a standardized checklist [24], which, in this case, was the CONTENT scale, as it was validated for the preoperative exercises [21].

### 4.2. Clinical Implications

Firstly, by exclusively focusing on high-risk patients who also benefited from different interventions, we addressed a current limitation in the literature regarding adequate selection of candidates. In this sense, 6-min walk test, Peak VO2 and different pulmonary function tests can be interesting options to identify early the optimum candidates, assess the patient’s response to the intervention, and track adherence during the program. In the second place, despite the heterogeneous results about the efficacy of aerobic training in previous reviews, we recommend its implementation in any of its forms as a core modality of physical prehabilitation, in line with the clinical practice guides for the pre-enrollment of physical exercise [25]. In the third place, we found that it was especially the studies lasting from one to four weeks and that offered multimodal programs (or, at least, multicomponent exercise therapy) that presented the most promising results. This can be useful to carefully identify the optimum interventions and eventually conduct more suitable programs adapted to high-risk patients awaiting cardiothoracic surgeries.

## 5. Conclusions

This systematic review enabled us to envisage the clinical implications of implementing different prehabilitation variants to improve the results obtained in the postoperative period in aged patients. A prehabilitation program lasting from one to four weeks seems effective in improving physical fitness and accelerate return to the preoperative functional state in aged patients subjected to cardiothoracic surgeries.

## Data Availability

Not additional data available.

## Supplementary Materials

The following are available online at https://www.mdpi.com/article/10.3390/healthcare9111602/s1, Table S1: Result of search strategy for each database; Table S2: Characteristics of the randomized clinical trials included; Table S3: Appraisal of the methodological quality of the studies selected; Table S4: Appraisal of the therapeutic validity of the studies selected.
