# Supplementary material for: Effects of Prehabilitation on Functional Capacity in Aged Patients Undergoing Cardiothoracic Surgeries: A Systematic Review
Source: Healthcare (Basel). 2021 Nov 22;9(11):1602. doi: 10.3390/healthcare9111602 (PMC8625473; doi:10.3390/healthcare9111602)
Supplement: Supplementary file 1 [file healthcare-09-01602-s001.zip › healthcare-1448194-supplementary.pdf]

healthcare

MDPI

Table S1. Result of search strategy for each database.

Databases	Dates	Search Strategy	Results
MEDLINE (via PubMed)	04/13/2021	(aged[Mesh] OR aged OR aging[Mesh] OR "aged, 80 and over" OR elder* OR elderly OR older* OR "Frailty"[Mesh] OR frail*) AND (Cardiothoracic OR "Cardiothoracic surgery" OR "Thoracic Surgery" OR "Cardiac Surgery" OR "Heart Surgery" OR "pulmonary surgical procedures" OR "Esophageal surgery" OR "Lung Transplantation" OR "Heart Transplantation") AND ("Preoperative Exercise" OR "Preoperative Rehabilitation" OR prehabilitation OR Prehab* OR "Pre-operative Conditioning" OR exercise OR "Exercise Therapy" OR "exercise training" OR "Physical Activity" OR "Physical Therapy" OR "Inspiratory muscle training" OR "Cardiopulmonary exercise" OR "Breathing Exercises" OR "Nutrition Therapy" OR "Cognitive Behavioral Therapy" OR "cognitive therapy") Filters: Randomized Controlled Trial, Humans	340
Web of Science	04/14/2021	(aged OR aged OR aging OR "aged, 80 and over" OR elder* OR elderly OR older* OR Frailty OR frail*) AND (Cardiothoracic OR "Cardiothoracic surgery" OR "Thoracic Surgery" OR "Cardiac Surgery" OR "Heart Surgery" OR "pulmonary surgical procedures" OR "Esophageal surgery" OR "Lung Transplantation" OR "Heart Transplantation") AND ("Preoperative Exercise" OR "Preoperative Rehabilitation" OR prehabilitation OR Prehab* OR "Pre-operative Conditioning" OR exercise OR "Exercise Therapy" OR "exercise training" OR "Physical Activity" OR "Physical Therapy" OR "Inspiratory muscle training" OR "Cardiopulmonary exercise" OR "Breathing Exercises" OR "Nutrition Therapy" OR "Cognitive Behavioral Therapy" OR "cognitive therapy") Refine to: Clinical Trial	441
Scopus	04/15/2021	#1: (aged OR aged OR aging OR "aged, 80 and over" OR elder* OR elderly OR older* OR frailty OR frail*) AND (cardiothoracic OR "Cardiothoracic surgery" OR "Thoracic Surgery" OR "Cardiac Surgery" OR "Heart Surgery" OR "pulmonary surgical procedures" OR "Esophageal surgery" OR "Lung Transplantation" OR "Heart Transplantation") AND ("Preoperative Exercise" OR "Preoperative Rehabilitation" OR prehabilitation OR prehab* OR "Pre-operative Conditioning" OR exercise OR "Exercise Therapy" OR "exercise training" OR "Physical Activity" OR "Physical Therapy" OR "Inspiratory muscle training" OR "Cardiopulmonary exercise" OR "Breathing Exercises" OR "Nutrition Therapy" OR "Cognitive Behavioral Therapy" OR "cognitive therapy")<br>#2: "Clinical Trial" OR Trial OR "Randomized controlled trial"<br>#3: Observational OR "case report" OR cohort OR "case control"<br>#4: #1 AND #2 AND NOT #3 (489). Limit to: articles	367

Table S2. Characteristics of the randomized clinical trials included.

Author/s and year	Participants	Training with Physical Exercise, including Other Prehabilitation Components				Result Measures	Main Findings
		Context/ Therapists	Intervention, by groups	Duration/ Frequency	Adherence		
Bathia and Kaiser [27], 2019	Pulmonary resection due to NSCLC, N, Mean Age (MA)=151, 64; IG, MA: n=74, 64 ± (13); CG, MA: n=77, 64 ± (10).	Respiratory care service, Geneva University Hospital (Switzerland); Respiratory Physiotherapists (PhTs).	Prehabilitation based on exercises. Supervised sessions. →IG*: Individualized HIIT sessions (Motion Cycle 500, Germany) adjusted by Borg scale > 5; 2 10-minute series interspersed by a 4-minute break: - Warming-up at 50% $Peak_W$- HIIT for 15 seconds at 100% $Peak_W$ interspersed by a 15-second break until reaching 10 minutes- 5-minute cooling-down at 30% $Peak_W$ → CG: Usual care (risk assessment, peri-operative blood and medication management, and hospital-related education).	2-4 weeks (median: 26 days, IQR: 21-33 days); 3 times/ week	87% ± 18 compliance (median: 8 sessions, Interquartile Range [IQR]: 7-10)	-Duration: Unknown. -Measurement: T1: Surgery decision; T2: Day before the surgery. -Aerobic / Physical exercise capacity [A, C]: $Peak_W$; Peak VO_2 -Functional capacity: 6MWT [A, C] At-rest Heart Rate (HR)[A, C] -HR after cooling-down [A, C]	The IG improved functional capacity by 20% (6MWT; 95% CI: 14-26%). -The IG improved aerobic capacity by 14% (Peak VO_2; 95% CI: 3-26%) and aerobic power by 7% ($Peak_W$; 95% CI: 2-13%); in addition, $HR_{at\ rest}$ was reduced by 6 bpm (95% CI: from -4 to -7 bpm). -In a subsequent analysis about this sample of patients (Licker et a l., 2017), HIIT also reduced pulmonary POCs and length of ICU stay.

Guinan et al. [32], 2019	Esophagectomy (transthoracic, n=43; hiatal n=17; *p*: 0.27) N, MA=60, 64.1 ± (7.8); IG, MA: n=28, 63 ± (8.8); CG, MA: n=32, 65 ± (7.7)	In the patients' homes. Non-supervised exercises. An initial training session by PhTs.	IMT-based prehabilitation. Non-supervised sessions.<br><br>→IG: IMT (*K3, POWERbreathe®)* 30 repetitions twice a day. Initially at 60% of baseline PIP, progressing 5% if Perceived Effort Rate (PER) < 7 (1-10 range).<br><br>→CG: Usual care. *Moderate AA was recommended in both groups*	2-3 weeks (mean: 21.5 ± 10.5 days)	Weekly phone follow-up and according to a training and registration diary. 5 patients did not complete the training session (n analyzed: 55). 64% compliance.	-Duration: 03/2014-05/2016.<br><br>-Measurement: T1: Prehab start; T2: Before the surgery; T3: Day 9 after the surgery.<br><br>-Physical activity level [D]: Minutes of activity/day and number of steps/days; (*Actigraph Pensacola, FL*)<br><br>-Functional capacity: 6MWT [D]<br><br>-Pulmonary function: FEV_1 [D]; FVC [D]; IMR [C]	-Substudy of the PREPARE RCT. -Moderate-level pre-operative AA: Greater in the CG (p=0.04).<br><br>-Pre-operative PIP: 68.37 cm H_2O in the IG and 59.20 cm H_2O in the CG (p<0.03). However, there was no difference with the CG 3, 6 and 9 days after the surgery.<br><br>-6MWT: 74.8 m less in the IG than in the CG before discharge (T3) (95% CI: 9.9-139.3 m, p<0.03).<br><br>-At day 1, the CG participants were more active, and walked more steps between day 1 and day 5 after the surgery than those in the IG (p<0.04).

Lai et al. [28], 2017	Pulmonary resection due to NSCLC, N, MA=101, 64.1 ± (7.4); *IG, MA*: n=51, 63.8 ± (8.2); *CG, MA*: n=50, 64.6 ± (6.6)	West China hospital rehabilitation room at full pre-operative hospitalization regime (Chengdu, China). PhTs and experienced nurses	Prehabilitation based on AT + IMT. Supervised sessions. →IG*. (a) Moderate AT for 30 minutes adjusted by SE (Borg scale > 6) and O_2 Sat < 88%. (b) Supervised IMT sessions: Deep breadths and incentive spirometry (HUDSON RCI 2500, USA), 20 repetitions/ session; and abdominal breadths during 15-30'/session. →CG: Usual care.	1-week, daily AT and IMT	89.4% compliance (n=2 did not tolerate intensity, n=2 did not notice any improvement, n=2 surgery was moved forward).	-Duration Unknown. -Measurement: T1: Prehab start, T2: Prehab end, 1 day before the surgery. -Functional capacity: 6MWT [A, C] -Pulmonary function: PEF [A, C] -QoL [B, D]: EORTC-QLQ-C30 -Dyspnea [B, D]: EORTC LC-13 -Pulmonary POCs [C] -PHT [C] -Health-related expenses [C]	-6MWT increased significantly in the IG, 22.9 ± 25.9 m, when compared to 4.2 ± 9.2 m in the CG (p<0.001). -PEF improved by 21 l/min in the IG (95% CI: 7.2–34.8; p=0.003). -QoL: Improvement in the IG and CG, although without differences (p=0.462). -Incidence of pulmonary POCs was reduced by 18.2% when compared to the CG (p=0.019). -Shorter overall PHT in the IG (15.6 ± 3.6 vs 17.7 ± 5.3 days, p=0.023).

Minnella et al. [33], 2018	Open esophagectomy, N, MA=51; 67.6; *IG, MA*: n=26, 67.3 ± (7.4); *CG, MA*: n=25, 68 ± (11.6)	In the patients' homes. Non-supervised exercises. An initial training session with a Kinesiologist linked to the Health Center of McGill University (Quebec, Canada)	Multimodal prehabilitation. Non-supervised sessions.<br><br>→IG*: (1) (a) Moderate AT (the patients were instructed to choose the intensity according to the Borg scale between 6 and 20):<br>-5-minute warming-up<br>-30 seconds at 12-13 of SE (Borg scale)<br>-5-minute cooling-down<br>(b) 3 series x 8-12 repetitions of MS for 8 muscle groups with an intensity of 5-6 out of 10 in the *ACSM/AHA* scale.<br>(2). Nutritional counseling + Protein supplementation (Immunocal; Immunotec Inc) for 1.2-1.5 g protein/Kg.<br><br>→CG: Usual care (*Enhanced recovery pathways for esophagectomy*)	2-10 weeks, 3 days/week for AT and MS	Weekly phone follow-up and according to a training and registration diary.<br>11 did not complete the post-operative assessment.<br>63% compliance.	-Duration: 02/2013-06/2017.<br><br>-Measurement: T1: Enrollment/Randomization, T2: Before the surgery, T3: 4-8 weeks after the surgery.<br><br>-Functional capacity: 6MWT [A, C]<br><br>-POCs (*Clavien Dindo, Comprehensive Complication Index*) [D]<br><br>-Mortality [D]<br><br>-PHT [D]	-The IG significantly improved functional capacity in the pre-operative (+36.9 m vs -22.5 m) and post-operative (+15.4 m vs -81.8 m) periods, as well as from baseline to discharge. In addition, in the IG, 52% attained functional improvements after the surgery.<br><br>-The number and severity of POCs, PHT and deaths were comparable between the groups.<br><br>-Better physical and nutritional conditions can significantly assist in undergoing systemic therapy or full cancer treatment.

Morano et al. [29], 2014	Pulmonary resection due to NSCLC, N, MA=24, 67; *IG, MA*: n=12, 65 ± (8); *CG, MA*: n=12, 69 ± (7)	Rehabilitation room of the Fortaleza hospitals (Brazil). PhTs.	Prehabilitation based on multicomponent training. No information about supervision.<br><br>→IG (pulmonary rehab group). (a) AT on treadmill at 80% Peak$_W$ Adjustment and length of sessions unknown. Follow-up and if there were training sessions unknown.<br>(b) with a bar at 50% of peak work capacity.<br>(c): IMT: Breathing patterns, breadths with pursed lips and incentive spirometer (*Respiron*®, *Brazil*).<br><br>→CG (thoracic physiotherapy group) underwent IMT training.	4 weeks, 5 days/week for all the exercises.	Follow-up and compliance rate unknown.	-Duration: 03/2008-03/2011.<br><br>-Measurement: T1: Surgery decision, T2: Prehab end (4 weeks after T1).<br><br>-Functional capacity: 6MWT [B, D], UULEX [A, C]<br><br>-QoL: SF-36 [B, D], HADS [A, C]<br><br>-[Fibrinogen in mg/dl] [A, C]<br><br>-[Albumin in g/dl] [B, D]	-Distance in 6MWT decreases after the intervention (from 425 ±85 m to 339 ± 108 m), and is not significant in relation to the CG; In addition, Strength in UULL increased from 1.13 to 1.58 kg per month, and with a mean of 0.7 in relation to the CG ($p<0.02$)<br><br>-The anxiety and depression scores are significantly reduced ($p<0.02$), the final QoL score is comparable to the CG ($p= 0.59$).<br><br>-Peri-operative PR is effective in reducing the fibrinogen blood levels, but not those of albumin.

Sebio et al.[31], 2017	VATS due to NSCLC, N, MA=22, 70.1; *IG, MA*: n=10, 70.9 ± (6.1); *CG, MA*: n=12, 69.4 ± (9.4)	Hospital rehabilitation room, Corunna (Spain). Respiratory PhTs.	Prehabilitation based on multicomponent training. Supervised sessions.<br><br>→IG*: (a) 30 minutes of moderate AA (*Monark 818 E* cycloergometer) determined by $Peak_w$<br>-5-minute warming-up (30% $Peak_w$)<br>-1 minute (80% $Peak_w$)<br>-4-minute active rest (30% $Peak_w$)<br>-4-minute cooling-down (30% $Peak_W$)<br>(b) MS (*Theraband*), 3 series (4 series if the patient tolerates it) x 15 repetitions for 6 muscle groups according to SE; 45-second rest between series.<br>(c) IMT (*Coach 2 Incentive Spirometer* ®) 6 series x 5 breadths at 80% of FVC; 1-minute rest between series.<br><br>→CG: It received IMT training.	7-8 weeks, 3-5 days for all the exercises	50% compliance (pre-IG: n=20, 2 abandoned PR (10%), 8 for other reasons; Final IG: n=10). Median of 16 (8-25) sessions.	-Duration: 2013-2015.<br><br>-Measurement: T1: Enrollment/Randomization, T2: Prehab end (median of 54.5 days), T3: 3 months after the surgery.<br><br>-Functional capacity: 6MWT [B, D], UULL strength (*Arm Curl Test*) [A, C] LLLL strength (*30′s Chair-to-Stand Test*) [A, C]<br><br>-Physical activity level [A, C]: Sustained length during CPET<br><br>-QoL: SF-36 v2 [A]<br><br>POCs (*Melbourne group*) [D]<br><br>-PHT [D]	-Between T1 and T2, the IG improved physical activity level (+397 s, p<0.001), overall health, PhC (+4.4 points, p=0.08) and muscle strength Improvement in 6MWT was not significant (+7.5 m, 95% CI -2.1 m-17.1 m).<br><br>-T3: there were no differences between the groups, although level of activity (+226 s vs -137 s, p<0.005), muscle strength (p=0.045 and p=0.002) and PhC (+4.3 vs -4.8 points, p=0.001) did present significant improvements; 6MWT in the IG remains practically the same when compared to the CG (p=0.186).

Stammers [35], 2016	CABG, *CABG + MVR* or *CABG + AVR*<br>N, MA=26, 71.5; *IG, MA*: n=14, 72.8 ± (7.1); *CG, MA*: n=12, 70.3 ± (5.4)	Community CR centers attached to the St. Boniface and St. James Hospitals, and Queen Elizabeth II HSC (Canada). PhTs and medical staff.	Multimodal prehabilitation. Supervised sessions.<br><br>→IG*. (1). MVPA sessions, previously adjusted and agreed upon by a specialized cardiologist:<br>-15-minute warming-up.<br>-10-30′ MVPA 40-60% HR (*Karvonen Formula*): 85% Peak VO_2, if the patient tolerates it.<br>-10-minute cooling-down.<br>(2). 4 psychosocial sessions (lifestyles, stress management and motivation).<br><br>→CG: Usual care. Low-intensity physical exercises were recommended.	7.7 (5.5) weeks, twice a week	Total program compliance (8 or more AT sessions and the 4 sessions) of 28.6% (n=4). 8 patients complied with at least 25% of the sessions. The mean of sessions attended/patient was	-Duration: 03/2015-01/2016.<br><br>Measurement: T1: After accepting to participate/ randomization (55-60 days before T2); T2: 1 week before the surgery; T3: 3 months after the surgery; T4: 1 year after the surgery.<br><br>-Frailty: FFP, FFI [A]<br><br>-Functional capacity [B, D]: 6MWT, SPPB<br><br>-Physical activity level: Sustained MVPA, in minutes [B, D].<br><br>-PHT [D]	-Substudy of the PREPARE multicentric RCT. The frailty, physical activity level and SPPB scores did not show differences in relation to the CG. 8 participants from the CG and 10 from the IG maintained moderate functional limitations at T1-T2 (SPPB < 9).<br>-Although 6MWT improved 10.3% when compared to the CG, it was not statistically significant (difference between the means: +34.2 m vs 4.6 m, $p>0.05$). If we perform an analysis by subgroups between the IG that completed the program and the partial prehab IG, the full-prehab patients improved by 35% ($p<0.05$) their FFI scores, whereas those in partial prehab worsened by 5%.<br>-Adherence to the program supposes greater benefits.<br>-PHT and length of ICU stay were

							comparable between the 2 groups. PHT (7[6.5-8.5] vs 8 [5-19.5] days, *p*>0.05).
Steinmetz et al. [34], 2019	CABG, N, MA=203, 67.1 ± 8,4; *IG, MA*: n=88, 66.1 ± (9); *CG, MA*: n=115, 67.9 ± (7.9).	Clinic's rehabilitation room (Ke-rckh off Heart Center, Germany). PhTs.	Prehabilitation based on multicomponent training. Supervised sessions.<br><br>→IG*:<br>(a) AA x 2 series/day at 70% Peak VO_2 with incremental time/session (1st sessio n 2 x 10′; 2nd and 3rd sessions 2 x 15′; 4th and 5th sessions 2 x 20′; 6th session 2 x 25′) with each session interspersed by 15′ of breathing + coordinatio n exercises.<br><br>→CG: Usual care.	2 weeks, 3 days/wee k	92% compliance (n of the CG at T1=88, n at T4=81; did not receive the intervention: 7, did not answer: 1).	-Duration: 12/2014-03/2018<br><br>Measurement:<br>T1: 2.5-3 weeks before CABG; T2: One day before; T3: CR (7-10 days after); T4: End of CR (3 weeks after).<br><br>-Aerobic capacity [B, D]: $Peak_W$, Peak VO_2<br><br>-Functional capacity [A, C]: 6MWT, TUG (in seconds)	- Cardiorespiratory capacity improves in the pre- and post-operative periods only in the IG, although without differences in relation to the CG.<br>-From baseline to 3 weeks after the surgery, 6MWT improved in both groups, although markedly more in the IG (6MWT; +50.5 m vs +14.5 m, p<0.001). TUG improved in time (T1-T4; -0.5 s, p<0.001) and in relation to the CG (difference of

						-QoL: *McNew* questionnaire [B, D]	mean at T4: -1.1 s, p=0.018). The improvements in relation to QoL were more accentuated in the IG than in the CG (p=0.137).
Vagvolgyi et al. [30], 2018	Pulmonary resection due to NSCLC, N, MA=238, 63.6; PRE group (IG), MA: n=72, 65 ± (7); Pre- and post-operative intervention group (RPP) (IG), MA: n=86, 65 ± (6); POST group (CG), MA: n=80, 61 ± (10).	PR room (Koranyi National Neumology Institute, Hungary). PhTs.	Prehabilitation based on AT and IMT. Supervised sessions. IG*. (1) (a) PRE group (AA-IMT) and RPP group (pre- AA-IMT + post- IMT). 2-3 series/day of customized AT in treadmill, with incremental intensity: 60% → 80% Peakw according to SE (the objective was to maintain a value of 7 in Borg scale). (b) 30 daily minutes of IMT: Controlled breathing, expectoration and thoracic mobilization. (2) 45-minute smoking cessation sessions and motivational and psychological support. Exact number of sessions unknown.	3 weeks. Weekly frequency of AT unknown. Daily IMT.	Adherence rates not reported, and follow-up unknown.	-Duration: 11/2016-01/2018.<br><br>-Measurement: T1: Prehab start (3 weeks before); T2: Prehab end, surgery; T3: Post-operative PR end (3 weeks after).<br><br>-Functional capacity: 6MWT [A].<br><br>-Physical activity level: Kilometers covered and sustained AA [A].<br><br>-Pulmonary function [A]: FEV, FVC, MIP<br><br>-QoL: mMRC [A]	-The 3 groups improved functional (6MWT) and aerobic (cycloergometry) capacity levels, pulmonary function and QoL after their respective interventions.<br><br>-RPP denoted the greatest improvements as it benefited from double PR, before and after. Many of these were significant in relation to the initial value, for example: 6MWT (T1-T3; from 378.3 m ± 90.5 to 403.3 m ± 98.4 m, p<0.0001) and time sustained low in CPET and distance covered (from 7.2 ± 3.2 to

	14.5 ± 4.5 minutes; from 3.6 ± 1.9 to 7.5 ± 2.9 km, p<0.0001).<br><br>-After a discriminant analysis of several measures, the authors show an effective algorithm to predict the risk of POCs, based on a combination and stratification of age, FEV_1, initial 6MWT value and distance covered in the treadmill.

Non-Small Cell Lung Cancer (NSCLC); High-Intensity Interval Training (HIIT); PeakW (Peak aerobic power); Aerobic Training (AT); Peak oxygen uptake (Peak VO2); 6-Minute Walk Test (6MWT); Cardiopulmonary Effort Test (CPET); Inspiratory Muscle Training (IMT); Peak Inspiratory Pressure (PIP); Forced Expiratory Volume in 1 second (FEV1); Forced Vital Capacity (FVC); Inspiratory Muscle Resistance (IMR); Subjective Exhaustion (SE); Peak Expiratory Flow (PEF); Post-operative Complications (POCs); Prolonged Hospitalization Time (PHT); Quality of Life (QoL); Muscle Strengthening (MS); Physical Component (PhC); Mental Component (MC); Lower Limbs (LLLL) and Upper Limbs (UULL); Unsupported Upper Limbs Effort Test (UULEX); Hospital Anxiety and Depression Scale (HADS); Short-form-36 health questionnaire (SF-36); Video-Assisted Thoracic Surgery (VATS); Coronary Artery Bypass Grafting (CABG); Aortic Valve Repair/Replacement (AVR) or Mitral Valve Repair/Replacement (MVR); Moderate-Vigorous Physical Activity (MVPA); Functional Frailty Index (FFI); Cardiac Rehabilitation (CR); Time-up and Go test (TUG); Chronic Obstructive Pulmonary Diseases (COPD); modified Medical Research Council scale (mMRC). A: Significant improvements in the IG between baseline and last measurement; B: No significant improvements in the IG between first and last measurements; C: Statistically significant improvement in the IG in relation to the CG; D: No significant differences between the IG and the CG; *: Content and intensity of the physical exercises were selected and adjusted according to theoretically argued recommendations.

Table S3. Appraisal of the methodological quality of the studies selected.

Author/s and Year	Scores of the Jadad Items								Risk of Bias/Limitations
	1	2	3	4	5	6	7	Total	
**Bathia and Kaiser [27], 2019**	1	1	0	0	1	0	0	3	-Risk of conduction bias, as there is no blinding of the participants.
**Guinan et al. [32], 2019**	1	1	0	0	1	0	0	3	-Risk of conduction bias, as there is no blinding of the participants.<br>-Significant differences in the level of moderate physical activity between the IG and the CG before the surgery.
**Lai et al. [28], 2017**	1	1	0	0	1	0	0	3	-Risk of conduction bias, as there is no blinding of the participants.
**Minnella et al . [33], 2018**	1	1	0	0	1	0	0	3	-Reduced sample size.<br>-Risk of conduction bias, as there is no blinding of the participants.
**Morano et al. [29], 2014**	1	1	0	0	1	0	0	3	-Reduced sample size.<br>-Risk of conduction bias, as there is no blinding of the participants.
**Sebio et al. [31], 2017**	1	1	0	0	1	0	0	3	-Reduced sample size.<br>-Risk of conduction bias, as there is no blinding of the participants.
**Stammers [35], 2016**	1	1	0	0	1	0	0	3	-Reduced sample size.<br>-Risk of conduction bias, as there is no blinding of the participants.
**Steinmetz et a l. [34], 2019**	1	1	0	0	1	0	0	3	-Risk of conduction bias, as there is no blinding of the participants.
**Vagvolgyi et al. [30], 2018**	1	1	0	0	1	0	0	3	-Risk of conduction bias, as there is no blinding of the participants.
**Methodological quality according to the Jadad scale (0-5): low (<3), acceptable (3), good (4) and excellent (5)**									

Table S4. Appraisal of the therapeutic validity of the studies selected.

**Author/s and Year**	**Selection of the Patients**	**Selection of the Therapist and of the Environment**		**Reason**		**Content**			**Adherence**	**Overall Score**
	**Description**	Adequate		Exercise based on objectives and hypothesis *a priori*	Content and intensity are described and valid	Intensity	Controlled and adjusted exercise	Customized		
**Bathia and Kaiser [27], 2019**	**Yes**	Yes	Yes	Yes	Yes	Yes	Yes	Yes	Yes	9 (100%)
**Guinan et al. [32], 2019**	No	Yes	No	Yes	No	Yes	Yes	No	No	4 (44.4%)
**Lai et al. [28], 2017**	Yes	Yes	Yes	Yes	Yes	Yes	Yes	Yes	Yes	9 (100%)
**Minnella et al. [33], 2018**	Yes	Yes	Yes	Yes	Yes	Yes	Yes	Yes	Yes	9 (100%)
**Morano et al . [29], 2014**	Yes	Yes	No	No	No	Yes	No	No	No	3 (33.3%)
**Sebio et al. [31], 2017**	Yes	Yes	Yes	Yes	No	Yes	Yes	No	Yes	7 (77.7%)
**Stammers [35], 2016**	Yes	Yes	Yes	Yes	Yes	Yes	Yes	Yes	No	8 (88.8%)
**Steinmetz et al. [34], 2019**	Yes	Yes	Yes	Yes	No	Yes	Yes	Yes	No	7 (77.7%)
**Vagvolgyi et al. [30], 2018**	Yes	Yes	No	Yes	No	No	No	No	No	3 (33.3%)
**Overall score**	8 (88.8%)	9 (100%)	6 (66.6%)	8 (88.8%)	3 (33.3%)	8 (88.8%)	7 (77.7%)	5 (55.5%)	4 (44.4%)	
